# Melanoma-associated Chondroitin Sulfate Proteoglycan (MCSP)-targeted delivery of soluble TRAIL potently inhibits melanoma outgrowth *in vitro *and *in vivo*

**DOI:** 10.1186/1476-4598-9-301

**Published:** 2010-11-23

**Authors:** Marco de Bruyn, Anna A Rybczynska, Yunwei Wei, Michael Schwenkert, Georg H Fey, Rudi AJO Dierckx, Aren van Waarde, Wijnand Helfrich, Edwin Bremer

**Affiliations:** 1 Surgical Research Laboratories, Department of Surgery, University Medical Center Groningen (Hanzeplein 1), University of Groningen, Groningen (9713 GZ), The Netherlands; 2 Department of Nuclear Medicine and Molecular Imaging, University Medical Center Groningen (Hanzeplein 1), University of Groningen, Groningen (9713 GZ), The Netherlands; 3 Third department of General Surgery, First Clinical Hospital of Harbin, Medical University Harbin (23 Youzheng Street), Harbin (150001), China; 4 Genetics, University of Erlangen Nuremberg (Erwin Rommel Strasse 3), Erlangen (91058), Germany

## Abstract

**Background:**

Advanced melanoma is characterized by a pronounced resistance to therapy leading to a limited patient survival of ~6 - 9 months. Here, we report on a novel bifunctional therapeutic fusion protein, designated anti-MCSP:TRAIL, that is comprised of a melanoma-associated chondroitin sulfate proteoglycan (MCSP)-specific antibody fragment (scFv) fused to soluble human TRAIL. MCSP is a well-established target for melanoma immunotherapy and has recently been shown to provide important tumorigenic signals to melanoma cells. TRAIL is a highly promising tumoricidal cytokine with no or minimal toxicity towards normal cells. Anti-MCSP:TRAIL was designed to **1**. selectively accrete at the cell surface of MCSP-positive melanoma cells and inhibit MCSP tumorigenic signaling and **2**. activate apoptotic TRAIL-signaling.

**Results:**

Treatment of a panel of MCSP-positive melanoma cell lines with anti-MCSP:TRAIL induced TRAIL-mediated apoptotic cell death within 16 h. Of note, treatment with anti-MCSP:sTRAIL was also characterized by a rapid dephosphorylation of key proteins, such as FAK, implicated in MCSP-mediated malignant behavior. Importantly, anti-MCSP:TRAIL treatment already inhibited anchorage-independent growth by 50% at low picomolar concentrations, whereas > 100 fold higher concentrations of non-targeted TRAIL failed to reduce colony formation. Daily i.v. treatment with a low dose of anti-MCSP:TRAIL (0.14 mg/kg) resulted in a significant growth retardation of established A375 M xenografts. Anti-MCSP:TRAIL activity was further synergized by co-treatment with rimcazole, a σ-ligand currently in clinical trials for the treatment of various cancers.

**Conclusions:**

Anti-MCSP:TRAIL has promising pre-clinical anti-melanoma activity that appears to result from combined inhibition of tumorigenic MCSP-signaling and concordant activation of TRAIL-apoptotic signaling. Anti-MCSP:TRAIL alone, or in combination with rimcazole, may be of potential value for the treatment of malignant melanoma.

## Background

Patients diagnosed with localized melanoma have a 10-year survival rate of up to 95%. In contrast, the life expectancy of patients with metastasized melanoma is limited to only 6 - 9 months [1]. The poor survival of patients with advanced melanoma is largely attributable to resistance towards current treatment modalities such as chemo- and radiotherapy [2]. Therefore, the development of novel therapeutic approaches that can trigger melanoma-selective cell death appears warranted.

One target molecule of potential relevance for the tumorigenicity of melanoma is the melanoma chondroitin sulfate proteoglycan (MCSP), also known as high molecular weight melanoma associated antigen (HMW-MAA). MCSP is highly expressed on the cell surface of both benign dysplastic nevi and on over 85% of malignant melanomas [3]. Importantly, over-expression of MCSP correlates with an unfavorable prognosis [4]. Expression of MCSP on normal tissue is mainly restricted to cells of the melanocyte lineage, but has also been detected in basal cells of the epidermis and within the hair follicle, in pericytes, chondrocytes and smooth muscle cells [3]. Recent studies have revealed that MCSP expression may provide important tumorigenic signals to melanoma cells. MCSP signaling stimulates growth, motility, and tissue invasion by melanoma cells, e.g. by enhancing integrin function [5], activation of Focal Adhesion Kinase (FAK) [6], mitogenic ERK signaling [7] and matrix metalloproteinase 2 [8]. Furthermore, non-metastatic radial growth tumor cells acquired anchorage-independent growth characteristics upon ectopic expression of MCSP [6]. Of note, anti-MCSP antibody treatment can partly inhibit MCSP-tumorigenic signaling *in vitro*, as evidenced by a pronounced inhibition of FAK [6].

Thus, MCSP appears to be important for melanoma tumorigenicity and appears to be a promising target for both naked monoclonal antibody (mAb) as well as immunotoxin-based strategies [9,10]. Notably, anti-MCSP mAbs proved to have beneficial effects on the clinical course of the disease of melanoma patients [3,11].

In recent years, we have demonstrated that scFv antibody fragment-targeted delivery of the immuno cytokine TRAIL holds particular promise for tumor-selective induction of apoptosis in various cancer types. TRAIL (Tumor Necrosis Factor Related Apoptosis Inducing Ligand) is a highly promising anti-cancer agent with pronounced pro-apoptotic activity towards various malignant cell types, including melanoma. Importantly, TRAIL essentially lacks activity towards normal cells [12]. Based on these characteristics, recombinant soluble TRAIL (sTRAIL) preparations have recently entered clinical trials, with promising preliminary reports on anti-tumor activity and safety [13].

Antibody fragment-mediated targeting of TRAIL can further selectively enhance the anti-tumor activity of TRAIL towards various types of cancer, including carcinomas and Acute Myeloid Leukemia [12,14-20]. Briefly, genetic fusion of TRAIL to a scFv antibody fragment allows for the selective delivery of TRAIL to a pre-selected tumor-associated antigen at the tumor cell surface. The resulting high levels of tumor cell surface bound TRAIL then efficiently activate apoptotic signaling via the agonistic TRAIL-receptors TRAIL-R1 and TRAIL-R2 in a mono- and/or bi/multicellular manner [14-18,20]. Of note, non-targeted sTRAIL has no intrinsic tumor-selective binding activity and is less efficient in cross-linking and activating TRAIL-R2 [21].

Here we preclinically evaluated the anti-melanoma activity of MCSP-targeted delivery of TRAIL, using fusion protein anti-MCSP:TRAIL. Anti-MCSP:TRAIL was designed to selectively bind to MCSP at the cell surface of melanoma cells and simultaneously inhibit tumorigenic signaling by MCSP. Once bound to MCSP-expressing melanoma cells, the anti-MCSP:TRAIL fusion protein can activate apoptotic TRAIL-signaling. Since TRAIL resistance has been reported for melanoma [22] we further evaluated a combinatorial strategy in which anti-MCSP:TRAIL treatment was combined with rimcazole. Rimcazole is a sigma receptor (σR) ligand currently in clinical trials for various cancers that has shown potent single-agent anti-tumor activity towards glioma and breast cancer [23,24]. Sigma ligand-based therapy may also be of value for the treatment of melanoma since the two subtypes of σ-Rs, σ-R1 and σ-R2 are strongly overexpressed in this tumor [25,26].

MCSP targeting with anti-MCSP:TRAIL indeed appeared to inhibit MCSP-signaling and activated TRAIL-apoptotic signaling in melanoma cells *in vitro *and *in vivo*. Furthermore, TRAIL-apoptotic signalling in melanoma cells can be further increased by combinatorial treatment with rimcazole. The dual anti-melanoma activity of anti-MCSP:TRAIL alone, or in combination with rimcazole, may be of interest for treatment of melanoma.

## Results and Discussion

### MCSP-restricted induction of apoptosis by anti-MCSP:TRAIL

We and others have previously demonstrated that selective delivery of the cytokine TRAIL by genetic fusion to an appropriate tumor-selective scFv antibody fragment significantly enhances the anti-tumor activity of TRAIL towards the corresponding type of cancer [14-20]. Here, we adapted this targeted approach to malignant melanoma by exploiting an anti-MCSP scFv antibody fragment that was derived from the well-established monoclonal antibody (mAb) 9.2.27. The resultant fusion protein, anti-MCSP:TRAIL, was equipped to selectively accrete at the cell surface of MCSP-positive cells only and subsequently trigger TRAIL-mediated apoptosis.

**Figure 1:**
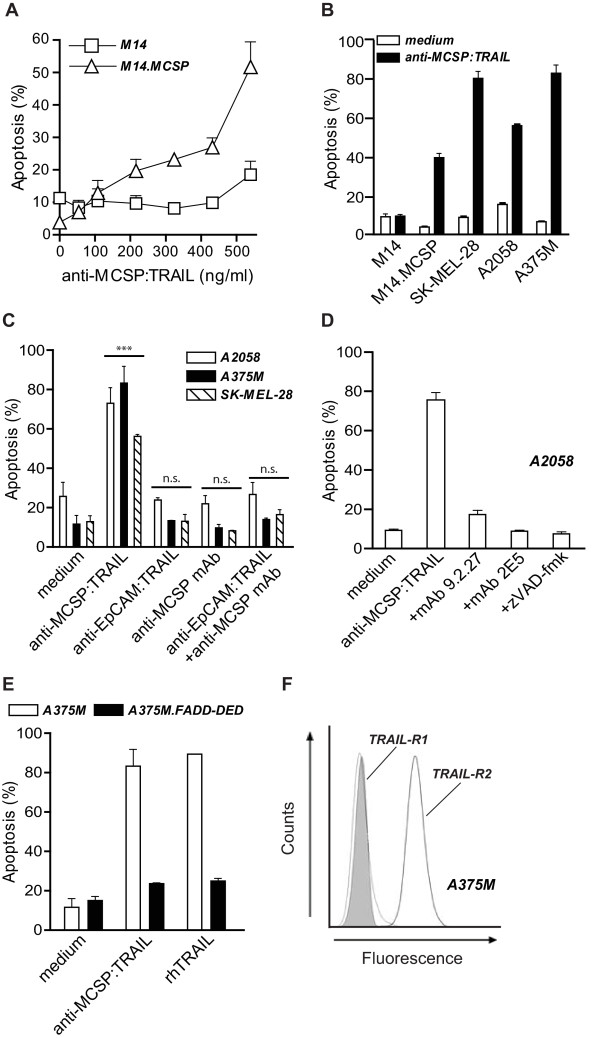
**MCSP-restricted induction of apoptosis by anti-MCSP:TRAIL**. **A **M14 and M14.MCSP cells were treated with increasing concentrations of anti-MCSP:TRAIL for 16 h and apoptosis was assessed by ∆ψ. **B **MCSP-negative cell line M14 and MCSP-positive cell lines M14.MCSP, SK-MEL-28, A2058 and A375M were treated with 500 ng/ml anti-MCSP:TRAIL for 16 h and apoptosis was assessed by ∆ψ. **C **A375M, A2058 and SK-MEL-28 cells were treated with equimolar concentrations of anti-MCSP:TRAIL, anti-EpCAM:TRAIL, anti-MCSP mAb or anti-EpCAM:TRAIL+anti-MCSP mAb for 16 h and apoptosis was assessed by ∆ψ. **D **A2058 cells were treated with 500 ng/mL anti-MCSP:TRAIL in the absence or presence of parental MCSP-blocking mAb 9.2.27, TRAIL-neutralizing mAb 2E5 or pan-caspase inhibitor zVAD-fmk for 16 h and apoptosis was assessed by ∆ψ. **E **A375M and A375M.FADD-DED cells were treated with anti-MCSP:TRAIL or rhTRAIL for 16 h and apoptosis was assessed by ∆ψ. **F **A375M cells were incubated with anti-TRAIL-R1 (thin line) or anti-TRAIL-R2 (thick line) mAb and expression of TRAIL-R1 and TRAIL-R2 was analyzed by flow cytometry. Shaded area indicates the fluorescence signal when cells were incubated with fluorescent-labeled secondary antibody alone.

Indeed, treatment of MCSP-transfected M14 melanoma cells (M14.MCSP) with anti-MCSP:TRAIL resulted in dose-dependent activation of apoptosis within 16 h, whereas parental MCSP-negative M14 cells were resistant to treatment with anti-MCSP:TRAIL (Figure 1A). Similarly, treatment of a series of MCSP-positive cell lines (A375M, A2058 and SK-MEL-28) with anti-MCSP:TRAIL resulted in a marked induction of apoptosis (Figure 1B). In a control experiment we treated the same series of melanoma cells with fusion protein anti-EpCAM:TRAIL, a fusion protein that is essentially identical to anti-MCSP:TRAIL except that it contains an anti-EpCAM scFv instead of an anti-MCSP scFv antibody fragment [14]. EpCAM is a well established carcinoma-associated cell surface target antigen that is not expressed on melanoma cells. Indeed, no signs of apoptosis were observed when A375M, A2058 or SK-MEL-28 cells were treated with anti-EpCAM:TRAIL (Figure 1C, Additional file 1, Figure S1A).

Treatment of MCSP-positive A375M, A2058 or SK-MEL-28 cells with the parental anti-MCSP antibody mAb 9.2.27 failed to induce any signs of apoptosis (Figure 1C). Importantly, also co-treatment with MAb 9.2.27 and anti-EpCAM:TRAIL did not induce any significant signs of apoptosis (Figure 1C). Thus, treatment of MCSP-positive melanoma cells with fusion protein anti-MCSP:TRAIL efficiently activates apoptosis that is MCSP-restricted and that is superior to combined treatment with TRAIL and the parental anti-MCSP mAb 9.2.27.

Induction of apoptosis by anti-MCSP:TRAIL was abrogated when treatment was performed in the presence of the parental mAb 9.2.27 (Figure 1D). Thus, anti-MCSP:TRAIL activity is strictly dependent on binding to cell surface-expressed MCSP on malignant cells. Furthermore, addition of the TRAIL-neutralizing mAb 2E5 fully abrogated the apoptotic effect of anti-MCSP:TRAIL (Figure 1D), indicating that induction of apoptosis is dependent on interaction of TRAIL with its agonistic TRAIL-receptors. In addition, induction of apoptosis was inhibited when treatment is performed in the presence of pan-caspase-inhibitor zVAD-fmk (Figure 1D), further suggesting involvement of TRAIL-mediated apoptotic signaling. Indeed, treatment of melanoma cells with anti-MCSP:TRAIL was characterized by time-dependent activation of initiator caspase-8 (Additional file 1, Figure S1B) as well as effector caspases 3 and 7 (Additional file 1, Figure S1C).

To further confirm that TRAIL-signaling is involved, the mutant cell line A375M.FADD-DED that ectopically overexpresses a dominant negative mutant of the adaptor protein FADD, was produced. FADD is a pivotal adaptor protein in TRAIL-receptor signaling. The mutant FADD protein lacks the so-called DED domain, which results in a general and strong resistance against TRAIL-mediated apoptosis. Indeed, TRAIL-mediated apoptotic signaling was fully halted in A375M.FADD-DED cells, as is evidenced by the fact that these cells were fully resistant to ubiquitously active recombinant human TRAIL (rhTRAIL) (Figure 1E). Likewise, A375M.FADD-DED cells were also fully resistant to treatment with anti-MCSP:TRAIL (Figure 1E). Taken together, these data clearly demonstrate that the pro-apoptotic activity of anti-MCSP:TRAIL is TRAIL-mediated.

Previously, we and others have shown that upon binding to the respective target antigen, scFv:TRAIL fusion proteins can efficiently activate TRAIL-R2, whereas untargeted sTRAIL preparations have a markedly reduced capacity to activate TRAIL-R2 signaling. Indeed A375M cells, that solely express TRAIL-R2 at the cell surface are sensitive to treatment with anti-MCSP:TRAIL (Figure 1B, Additional file 1, Figure S1E), indicating that anti-MCSP:TRAIL can potently activate TRAIL-R2 signaling in MCSP-positive melanoma cells. This feature may be of special relevance for melanoma since TRAIL-R2 is the most prevalent agonistic TRAIL-receptor expressed on melanoma cells [27].

### Potent inhibition of anchorage-independent growth by anti-MCSP:TRAIL

**Figure 2:**
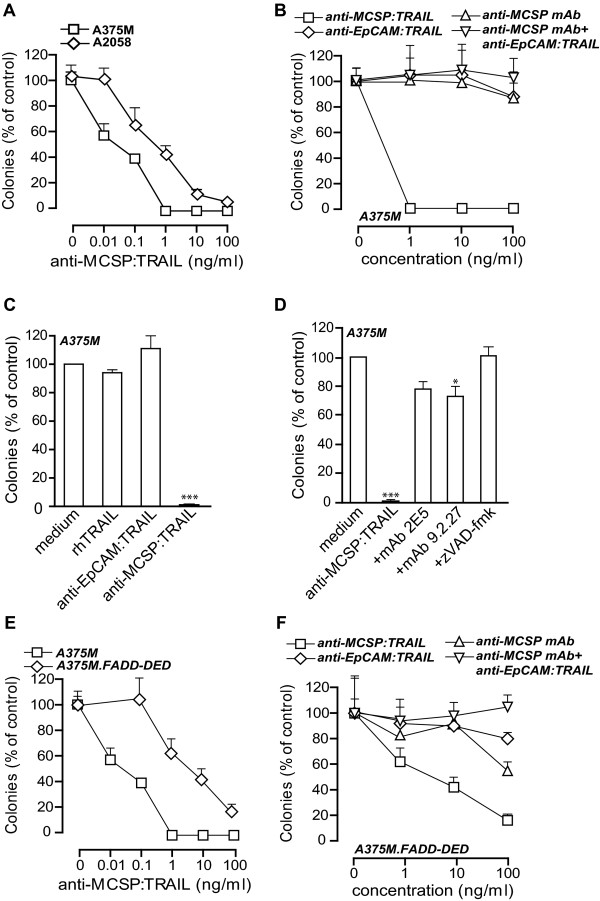
**Inhibition of anchorage-independent growth by anti-MCSP:TRAIL**. **A **A375M and A2058 cells were treated with increasing concentrations of anti-MCSP:TRAIL for 21 days and colony formation was assessed. **B **A375M cells were treated with increasing concentrations of anti-MCSP:TRAIL, anti-EpCAM:TRAIL, anti-MCSP mAb or anti-EpCAM:TRAIL+anti-MCSP mAb for 21 days and colony formation was assessed. **C **A375M cells were treated with rhTRAIL, anti-EpCAM:TRAIL or anti-MCSP:TRAIL for 21 days and colony formation was assessed. **D **A375M cells were treated with 100 ng/ml anti-MCSP:TRAIL in the absence or presence of TRAIL-neutralizing mAb 2E5, parental anti-MCSP mAb 9.2.27 or pan-caspase inhibitor zVAD-fmk for 21 days and colony formation was assessed. **E **A375M and A375M.FADD-DED cells were treated with increasing concentrations of anti-MCSP:TRAIL for 21 days and colony formation was assessed. **F **A375M.FADD-DED cells were treated with increasing concentrations of anti-MCSP:TRAIL, anti-EpCAM:TRAIL, anti-MCSP mAb or anti-EpCAM:TRAIL+anti-MCSP mAb for 21 days and colony formation was assessed.

The MCSP antibody fragment used in this study was derived from mAb 9.2.27. In earlier studies, mAb 9.2.27 partly inhibited MCSP-tumorigenic signaling *in vitro*, as evidenced by inhibition of FAK [9]. Since ectopic expression of MCSP was also recently shown to induce anchorage-independent growth capacity, anti-MCSP:TRAIL was evaluated for its effect on anchorage-independent growth of melanoma cells. In a soft agar assay, treatment of the MCSP-positive cell lines A375M and A2058 cells with anti-MCSP:TRAIL abrogated colony outgrowth in a dose-dependent manner (Figure 2A). Of note, anti-MCSP:TRAIL reduced colony formation by 50% already at very low concentrations (0.1 ng/ml and 1ng/ml for A375M and A2058 cells, respectively). Induction of apoptosis by anti-MCSP:TRAIL at these concentrations in adherent growth conditions was marginal (see Additional file 1, Figure S1E for dose-response curves of apoptosis induction). In contrast, treatment with similar concentrations of anti-EpCAM:TRAIL or with anti-MCSP mAb 9.2.27 only marginally reduced colony formation (Figure 2B). Importantly, also combinatorial treatment with equimolar concentrations of anti-EpCAM:TRAIL and mAb 9.2.27 failed to significantly inhibit colony outgrowth of A375M cells at the highest concentrations tested (Figure 2B). Furthermore, rhTRAIL failed to significantly reduce colony outgrowth (Figure 2C). Thus, anti-MCSP:TRAIL uniquely and efficiently prevents colony outgrowth.

Treatment of A375M cells with anti-MCSP:TRAIL in the presence of the TRAIL-neutralizing mAb 2E5 largely blocked the effect of anti-MCSP:TRAIL on colony outgrowth (Figure 2D). Similarly, treatment of cells with anti-MCSP:TRAIL in the presence of the parental anti-MCSP mAb 9.2.27 inhibited anti-MCSP:TRAIL activity, although the number of colonies still remained significantly lower than medium control (Figure 2D). Of note, co-incubation with pan-caspase inhibitor zVAD-fmk abrogated the inhibitory effect of anti-MCSP:TRAIL on colony outgrowth (Figure 2D), suggesting that apoptotic signaling is required for the inhibitory effect of anti-MCSP:TRAIL.

To further evaluate whether TRAIL-induced caspase-signaling was required for the inhibitory effect of anti-MCSP:TRAIL on colony outgrowth, TRAIL-resistant A375M.FADD-DED cells were treated with anti-MCSP:TRAIL. Importantly, colony formation of A375M.FADD-DED cells was inhibited by anti-MCSP:TRAIL treatment although ~50-100 fold higher concentrations were required to obtain the same magnitude of inhibition as observed for parental A375M cells (Figure 2E). Of note, treatment of A375M.FADD-DED cells with anti-EpCAM:TRAIL did not inhibit colony formation (Figure 2F), nor did combination treatment of anti-EpCAM:TRAIL and mAb 9.2.27 (Figure 2F). In contrast, mAb 9.2.27 alone did significantly inhibit colony outgrowth in this cell line, although to a lesser extent than anti-MCSP:TRAIL (Figure 2F). Together, these experimental data suggest that the simultaneous interaction of anti-MCSP:TRAIL with MCSP and TRAIL-receptors uniquely blocks anchorage-independent growth of melanoma cells at low concentrations.

### Anti-MCSP:TRAIL dephosphorylates proteins involved in cell proliferation and apoptosis resistance

**Figure 3:**
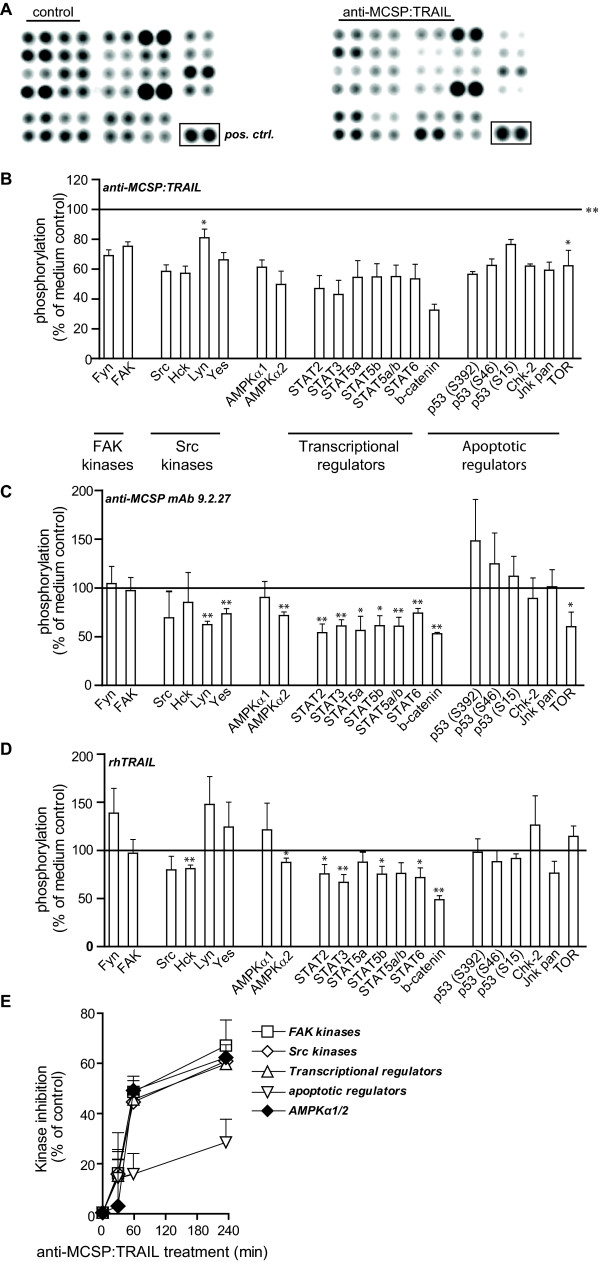
**Anti-MCSP:TRAIL triggers dephosphorylation of cellular proteins**. A375M cells were treated with anti-MCSP:TRAIL, anti-MCSP mAb 9.2.27, rhTRAIL or left untreated for 1 h. Whole cell lysates were analyzed for phosphorylation of 48 cellular kinases. **A **Phosphorylation of 28 kinases in untreated cells (left panel) or anti-MCSP:TRAIL-treated cells (right panel) are depicted. Images are representative of three independent experiments. **B, C, D **Changes in the phosphorylation of 21 kinases after treatment with **B **anti-MCSP:TRAIL, **C **anti-MCSP mAb 9.2.27 or **D **rhTRAIL are expressed as a percentage of the phosphorylation measured in untreated cells. **E **A375M cells were treated with anti-MCSP:TRAIL for 0, 30, 60 or 240 min. Whole cell lysates were analyzed for phosphorylation of 48 cellular kinases. Changes in phosphorylation of the different kinase groups as indicated in panel B are depicted. *, p < 0.05; **, p < 0.001; ***, p < 0.0001.

The efficacy of anti-MCSP:TRAIL on inhibition of colony formation by melanoma cells in 3D-culture suggests that antibody fragment-dependent sensitization of cells to concurrent TRAIL-signaling may contribute to its therapeutic effect. Previously, we generated proof of concept data for such dual therapeutic signaling with scFv:TRAIL fusion proteins, using an scFv:TRAIL fusion protein that targeted the Epidermal Growth Factor Receptor [16,19]. Here, the EGFR-inhibitory antibody fragment blocked mitogenic EGFR-signaling and synergized TRAIL apoptotic signaling [16,19]. To assess whether the MCSP-specific antibody fragment might contribute to the anti-tumor activity of anti-MCSP:TRAIL, a focused set of 48 cellular phosphoproteins was examined for the level of phosphorylation after treatment with anti-MCSP:TRAIL (Figure 3A B), anti-MCSP mAb 9.2.27 (Figure 3C) and rhTRAIL (Figure 3D). Interestingly, although A375M cells were treated with anti-MCSP:TRAIL in adherent growth conditions in view of experimental limitations with 3D-cultures, significant dephosphorylation of various proteins involved in cell survival and proliferation was detected after 1 h of treatment (Figure 3A, B and Additional file 2, Table S1). This dephosphorylation induced by anti-MCSP:TRAIL was detectable within 30 minutes and increased for up to 4 h after start of treatment (Figure 3E). Treatment with mAb 9.2.27 and rhTRAIL also triggered dephosphorylation of proteins at 1 h of treatment, but to a lesser extent and in a smaller panel (Figure 3C and 3D, respectively).

First and foremost, the previously reported down-stream target of MCSP, FAK, was dephosphorylated by a factor 2 compared to medium control. The parental anti-MCSP mAb 9.2.27 has been shown to prevent association of MCSP with integrins, thereby preventing activation of FAK [6]. Thus, although not determined here for anti-MCSP:TRAIL, a similar inhibition of MCSP/integrin interaction by the mAb 9.2.27-derived antibody fragment may be responsible for the inhibitory effect of anti-MCSP:TRAIL on FAK. Intriguingly, cross-linking of MCSP by bead-coated mAb 9.2.27 has also been used to achieve activation of MCSP-signaling [6]. The different outcome of treatment with soluble vs. coated mAb suggests that the extent of cross-linking of MCSP by the antibody determines the activation or inhibition, respectively, of downstream signaling. It will be interesting to evaluate in future studies whether the three MCSP reading heads in trimeric anti-MCSP:TRAIL are perhaps better suited for inhibition of MCSP-signaling.

The activity of ERK1/2, another established downstream effector of MCSP signaling [6], was not affected after 1 h of treatment with anti-MCSP:TRAIL. One possible reason for this finding is a perhaps delayed effect of anti-MCSP:TRAIL on ERK1/2 signaling at later and not examined time-points. In addition, it has been shown that MCSP enhances FAK and Erk1/2 signaling by distinct mechanisms [6]. It is therefore also conceivable that FAK is dephosphorylated while Erk1/2 is not a target of inhibition by anti-MCSP:TRAIL.

In addition to the established MCSP-target FAK, a panel of other proteins was dephosphorylated upon anti-MCSP:TRAIL treatment, including the kinase Fyn, and the Src kinases Src, Hck, Lyn and Yes. The relative impact of these respective proteins on MCSP tumorigenic signaling is currently being evaluated in extended ongoing studies using e.g. constitutively active and/or dominant negative mutants as well as small inhibitory RNA-mediated silencing of the individual components. Importantly, the above-mentioned experiments need to be performed not only in 2D, but also in 3D-cultures, in order to reliably determine the relative importance of the here identified proteins for MCSP-dependent anchorage-independent growth of melanoma cells.

Perhaps counter intuitively, the proto-oncogene Beta-Catenin (β-Catenin) was also dephosphorylated by treatment with anti-MCSP:TRAIL, as well as by treatment with mAb 9.2.27 and rhTRAIL (Figure 3B,C and 3D, respectively). Dephosphorylation of β-Catenin does not inactivate β-Catenin but actually prevents proteasomal degradation and increases cellular levels of β-Catenin. In turn, this leads to activation of pro-oncogenic gene transcription [28]. However, the exact role of β-Catenin-induced gene transcription in melanoma is still a matter of debate. Based on mouse models with activating mutations in β-Catenin, an oncogenic role of β-Catenin in melanoma was proposed [29]. On the other hand, others have shown that β-Catenin induces a transcriptional profile in melanoma cells that is reminiscent of normal melanocyte differentiation [30]. This transcriptional profile is moreover associated with increased patient survival and is lost upon malignant progression [30].

Taken together, anti-MCSP:TRAIL dephosphorylates a panel of established MCSP targets as well as newly identified proteins (see Additional file 2, Table S1 for overview). Thus, the kinase array data support a dual anti-melanoma activity by anti-MCSP:TRAIL that partly relies on inhibition of tumorigenic signaling by the anti-MCSP antibody fragment.

### Anti-tumor activity of low dose anti-MCSP:TRAIL towards A375M xenografts

**Figure 4:**
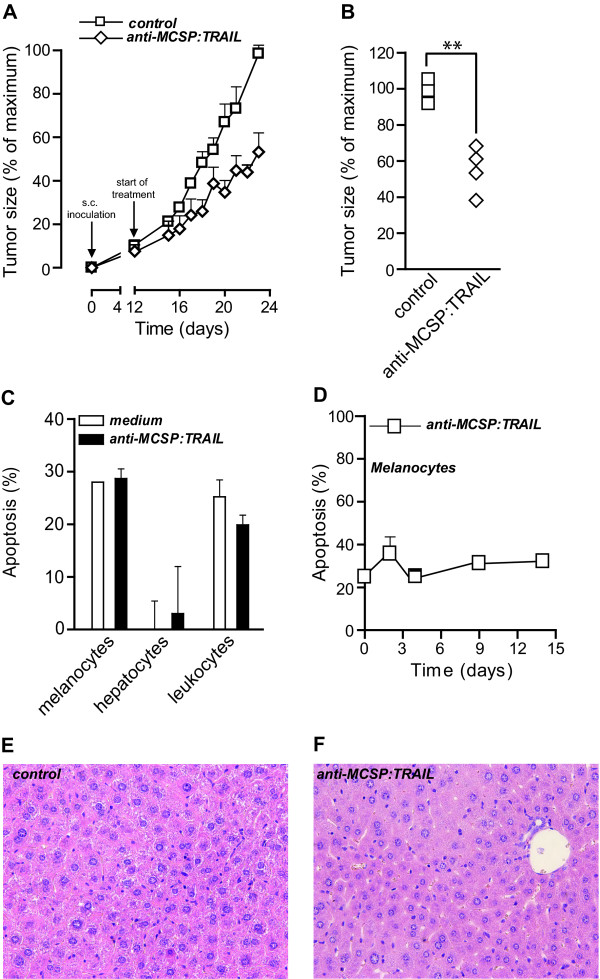
**Anti-tumor activity of anti-MCSP:TRAIL towards A375M xenografts**. **A **Mice were inoculated with A375M tumor cells at day 0 and developed xenografts of ~ 50 mm^3 ^after 12 days. Starting at day 12, mice were injected daily with saline (n = 4) or anti-MCSP:TRAIL (0.14 mg/kg, n = 4) and tumor size was measured daily using electronic caliper measurements. **B **Tumor size at day 23. **C **MCSP-positive melanocytes and MCSP-negative leukocytes and hepatocytes were treated with 500 ng/ml anti-MCSP:TRAIL for 16 h and apoptosis was assessed by ∆ψ. **D **Melanocytes were treated with 4 μg/mL anti-MCSP:TRAIL for up to 14 days and apoptosis was assessed by ∆ψ at time points indicated. **E **Liver pathology of mice carrying A375M xenografts was examined for Sham-treated mice or **F **anti-MCSP:TRAIL-treated mice.

To further characterize the anti-melanoma activity of anti-MCSP:TRAIL, A375M cells were xenografted subcutaneously in nude mice and allowed to form small tumors (~ 50 mm^3^). Subsequently, mice were treated daily by intra-peritoneal injection with a low dose of anti-MCSP:TRAIL (~ 0.14 mg/kg) or with saline. Of note, anti-MCSP mAb 9.2.27 and therefore anti-MCSP:TRAIL do not cross-react with mouse MCSP [3]. Compared to sham-treated mice, tumor size of the anti-MCSP:TRAIL-treated mice was strongly retarded in time (Figure 4A), with a 50% reduction in tumor size at the end of the experiment (Figure 4B). Of note, earlier animal studies with A375M cells and non-targeted rhTRAIL were performed at > 300 times higher concentrations (50 mg/kg) compared to the here employed treatment regimen with anti-MCSP:TRAIL [31]. Thus, in analogy to the low dose required for therapeutic activity in colony formation assays, anti-MCSP:TRAIL already has potent *in vivo *anti-melanoma activity at a very low dose.

### Anti-MCSP:TRAIL lacks apoptotic activity towards normal cells

Earlier studies have shown that tumor targeted delivery of TRAIL augments the tumor-specific activity of TRAIL, while not affecting the absence of toxicity on normal human cell types. Indeed, although anti-MCSP:TRAIL strongly bound to MCSP on normal human melanocytes (Additional file 1, Figure S1D), no apoptosis was induced in melanocytes even when treatment was performed with 4 μg/ml anti-MCSP:TRAIL (Figure 4C). Also extended treatment (up to 8 days) of melanocytes with 4 μg/ml anti-MCSP:TRAIL did not yield significant increases in apoptosis compared to medium control (Figure 4D). Similarly, normal human MCSP-negative hepatocytes were fully resistant to treatment with anti-MCSP:TRAIL (Figure 4C). Hepatocytes have previously been shown to be one of the most vulnerable normal cell types to possible TRAIL-related toxicity [32]. Importantly, also in nude mice carrying A375M xenografts treatment with anti-MCSP:TRAIL had no deleterious effect on liver morphology, with morphology of liver sections comparable to the morphology of liver sections of Sham-treated mice (Figure 4E and 4F). The here obtained data regarding the absence of activity of anti-MCSP:TRAIL towards normal cell types are in line with the large body of evidence in literature on the preclinical safety profile of sTRAIL. Indeed, ongoing clinical trials with rhTRAIL confirm the relative safety of TRAIL treatment with no serious adverse effects and no dose-limiting toxicity reported to date.

### Synergistic induction of apoptosis by anti-MCSP:TRAIL and σ-ligands

**Figure 5:**
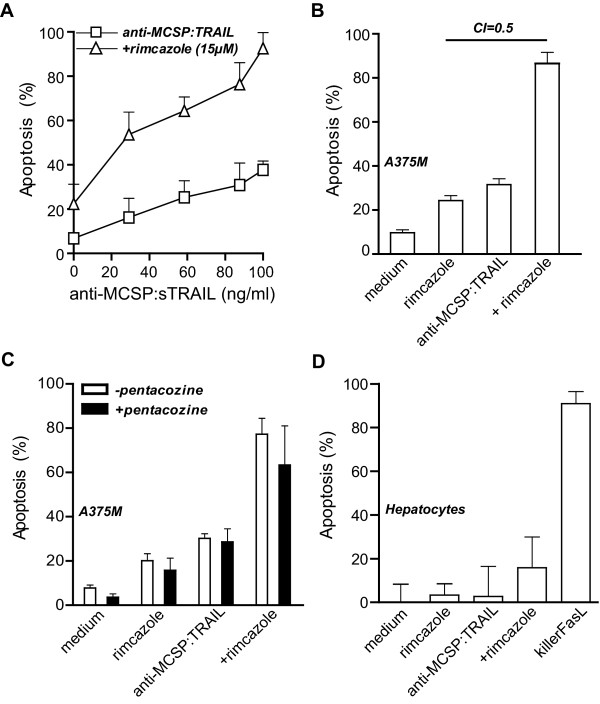
**Synergistic induction of apoptosis by anti-MCSP:TRAIL and rimcazole A**. A375M cells were treated with increasing concentrations of anti-MCSP:TRAIL in the presence or absence of rimcazole for 16 h and apoptosis was assessed by ∆ψ. **B **A375M cells were treated with rimcazole (15 μM), anti-MCSP:TRAIL (100 ng/ml) or both for 16 h, apoptosis was assessed by ∆ψ and the cooperativity index (CI) was calculated as indicated in the materials and methods section. **C **A375M cells were treated with rimcazole (15 μM), anti-MCSP:TRAIL (100 ng/mL) or both in the presence or absence of pentacozine (200 nM) for 16 h and apoptosis was assessed by ∆ψ. **D **Hepatocytes were treated with, rimcazole (15 μM), anti-MCSP:TRAIL (100 ng/ml) or both for 16 h and apoptosis was assessed by ∆ψ. KillerFasL (100 ng/ml)was used as a positive control for hepatocyte toxicity.

Since resistance of melanoma cells towards sTRAIL has previously been reported [22] a combinatorial strategy was evaluated of anti-MCSP:TRAIL with rimcazole, a σ-ligand in clinical trials for various cancers. Sigma receptors are expressed in the central nervous, immune, endocrine, and reproductive systems, but also in peripheral organs like kidney, liver and gastrointestinal tract. Although the precise function of these receptors remains unknown, both σ-R1 and σ-R2 are strongly overexpressed in rapidly proliferating cells such as melanoma and may be exploited as possible targets for melanoma therapy [26]. Treatment of A375M cells with the σ-R1/σ-R2 antagonist rimcazole synergistically enhanced induction of apoptosis by anti-MCSP:TRAIL (Figure 5A and 5B). Of note, treatment in the presence of the σ-R1 agonist (+)pentazocine did not abrogate synergy, suggesting that rimcazole synergizes with anti-MCSP:TRAIL activity via σ-R2 (Figure 5C). Rimcazole did not upregulate TRAIL-R1 or TRAIL-R2 expression (data not shown), but augmented the activation of initiator caspase-8 and initiator caspase-9 upon anti-MCSP:TRAIL treatment (Additional file 3, Figure S2A and B, respectively). Moreover, inhibition of either initiator caspase abrogated cytotoxic activity (Additional file 3, Figure S2C). Thus, rimcazole appears to synergize the activity of anti-MCSP:TRAIL by promoting caspase-8 activation. Of note, anti-MCSP:TRAIL in combination with rimcazole lacked apoptotic activity towards hepatocytes (Figure 5D), suggesting that this combination retains the favorable toxicity profile associated with TRAIL and rimcazole alone.

## Conclusions

**Figure 6:**
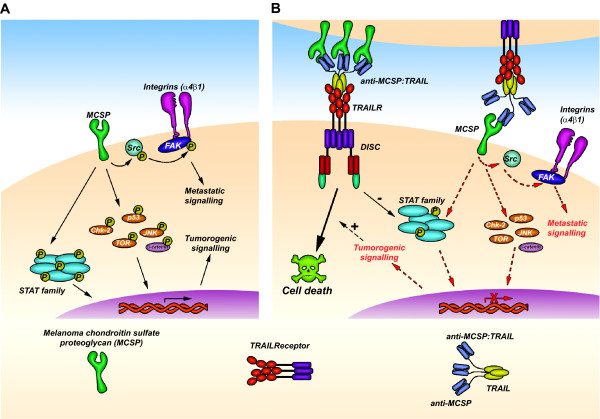
**Schematic representation of the anti-melanoma activity of anti-MCSP:TRAIL**. **A **Direct or indirect MCSP-mediated signaling involves (integrin-dependent) FAK and Src phosphorylation and maintains phosphorylation of downstream transcription factors and apoptotic modulators. MCSP thus contributes to pro-survival and metastatic signals. **B **Binding of anti-MCSP:TRAIL inhibits MCSP-mediated signaling and concomitant activation of pro-metastatic/-survival signals and thereby sensitizes cells to apoptosis induction via binding of the TRAIL moiety to TRAILR.

We provide evidence that anti-MCSP:TRAIL, a TRAIL fusion protein targeted to melanoma-expressed MCSP, inhibits MCSP tumorigenic signaling and simultaneously induces TRAIL apoptotic signaling. Consequently, fusion protein anti-MCSP:TRAIL potently inhibits outgrowth of melanoma cells both *in vitro *and *in vivo *and this effect can be further enhanced with the cytotoxic agent rimcazole. Based on the above, we postulate the following working model for anti-MCSP:TRAIL (see also Figure 6B); anti-MCSP:TRAIL binds to MCSP and inhibits MCSP-signaling, which includes inhibition of src-kinases and FAK, the STAT transcription factors, and various anti-apoptotic modulators such as p53, TOR, JNK. Concurrently, interaction of the sTRAIL domain with its agonistic receptors TRAIL-R1 and TRAIL-R2 triggers potent induction of apoptosis. Notably, TRAIL/TRAIL-R interaction also triggers dephosphorylation of the STAT family, which may contribute to sensitization of cells to apoptosis [33,34]. Thus, melanoma cells are eliminated on the one hand by MCSP-mediated sensitization of melanoma cells to apoptosis and on the other hand by the concurrent activation of TRAIL-apoptotic signaling. Taken together, anti-MCSP:TRAIL is a novel immunotherapeutic agent that, either alone or in combination with rimcazole, may be of potential value for treatment of advanced melanoma.

## Methods

### Reagents

MAb 9.2.27 is a murine IgG2a with high binding affinity for human MCSP [10]. TRAIL-neutralizing mAb 2E5 was purchased from Alexis (10P's, Breda, The Netherlands). Caspase inhibitors zVAD-fmk and zLEHD-fmk were from Calbiochem (VWR International B.V., Amsterdam, The Netherlands) and dissolved at 10 mM in DMSO. Rimcazole (BW 239U) and (+)pentazocine were from Sigma Aldrich (Sigma-Aldrich Chemie B.V. Zwijndrecht, Netherlands). Stock solutions of rimcazole (3.9 mM in ethanol) and pentazocine (10 mM in 0.1 N hydrochloric acid) were freshly prepared for each experiment.

### Cell lines

MCSP-positive/EpCAM-negative melanoma cell lines A375M, A2058 and SK-MEL-28 were obtained from and characterized (STR profiling, karyotyping, isoenzyme analysis) by the American Tissue Culture Collection (ATCC). The MCSP-negative melanoma cell line M14 and transfectant cell line M14.MCSP were provided by Prof. Dr. G.H. Fey. Expression of MCSP was determined by flow cytometry using anti-MCSP mAb 9.2.27 and a FITC-conjugated goat-anti-mouse mAb. A375M.FADD-DED cells were generated by transfection of parental A375M cells using Fugene (Roche BV, Woerden, The Netherlands). All cell lines were cultured at 37°C, in a humidified 5% CO_2 _atmosphere. A375M, A2058, SK-MEL-28 and M14 cells were cultured in RPMI 1640 medium (Cambrex Bio Science, Verviers, France) supplemented with 10% fetal calf serum. M14.MCSP cells were cultured in RPMI 1640 medium supplemented with 10% fetal calf serum and 400 μg/ml Geneticin. A375M.FADD-DED cells were cultured in RPMI 1640 medium supplemented with 10% fetal calf serum and 500 μg/ml Geneticin.

### Primary human hepatocytes (PHH) and melanocytes

Cryopreserved human hepatocytes and melanocytes (Tebu-bio bv, Heerhugowaard, The Netherlands) were isolated according to standard protocol using hepatocyte isolation kit and melanocyte isolation kit, respectively (tebu-bio bv, Heerhugowaard, The Netherlands). For experiments, hepatocytes and melanocytes were seeded in a 48-well plate at a density of 0.5 × 10^6 ^cells/ml.

### Construction of fusion protein anti-MCSP:TRAIL

A scFv antibody fragment in the VH-VL format was derived from mAb 9.2.27 using standard antibody-phage display technology [10]. Fusion protein anti-MCSP:TRAIL was constructed by cloning the cDNA of antibody fragment MCSP in frame with soluble TRAIL into in-house constructed vector pEE14 using unique SfiI and NotI restriction enzyme sites, yielding plasmid pEE14-anti-MCSP:TRAIL. Fusion protein anti-MCSP:TRAIL was produced in CHO-K1 cells using previously described methods [14,16]. Culture medium containing anti-MCSP:TRAIL was cleared by centrifugation (10 000 g, 10 min), filter sterilized, and stored at -80°C until further use. Fusion protein anti-MCSP:TRAIL was purified via the N-terminal Hemagglutinin (HA)-tag using anti-HA affinity chromatography. Fusion protein scFvC54:sTRAIL [14], for reasons of clarity hereafter renamed into anti-EpCAM:TRAIL, is a fusion protein that is essentially identical to anti-MSCP:TRAIL except that it contains an anti-EpCAM scFv instead of an anti-MSCP scFv.

### Assessment of apoptosis

MCSP-restricted induction of apoptosis by anti-MCSP:TRAIL was assessed on a panel of melanoma cell lines. Briefly, melanoma cells were pre-cultured in a 48-well plate at a concentration of 3 × 10^4 ^cells/well. Subsequently, cells were treated as indicated and apoptosis was assessed by: **Loss of mitochondrial membrane potential (∆ψ); **∆ψ was analyzed by DiOC6 staining (Eugene, The Netherlands) as previously described [16]. After 16 h treatment, cells were harvested and incubated for 20 min with DiOC6 (0.1 μM) at 37°C, harvested (1000 g, 5 min.), resuspended in PBS, and assessed for staining by flow cytometry. **Caspase-8, caspase-9 and caspase-3/-7 activity; **caspase activity was assessed using the Caspase-Glo 8, 9 and 3/7 Assay according to manufacturer's instructions (Promega Benelux BV, Leiden, The Netherlands). The assay is based on the cleavage of non-luminescent substrates by activated caspases into a luminescent product. Luminescence was quantified using a Victor3 multi-label plate reader (Perkin Elmer, Groningen, The Netherlands)

### Soft agar colony forming assay

Soft agar colony forming assays were performed in 24-well plates pre-coated with 0.5 ml solidified 0.4% agarose in RPMI 1640 medium. Cells were resuspended at a density of 10 × 10^3 ^cells/well in 0.6% agarose in RPMI 1640 medium supplemented with 20% fetal calf serum and layered on the solidified 0.4% agarose in a 24-well plate. Tumor cell containing agarose was allowed to solidify for 10 minutes at 4°C before addition of 1 ml of RPMI 1640 medium supplemented with 20% fetal calf serum. Cells were treated as indicated and subsequently cultured at 37°C, in a humidified 5% CO_2 _atmosphere. Colony formation was assessed 21 days after start of treatment. Assays were performed in quadruplicate. Number of colonies was quantified and percentage of colony growth was calculated with the formula: Percentage of colony growth = (number of colonies in experimental condition)/(number of colonies in medium control) × 100%. For A375M cells, the number of colonies in untreated samples ranged from 30 ± 5 to 262 ± 35 between individual experiments.

### Proteome Profiler array

The effect of MCSP targeting on protein tyrosine kinase activity was assessed by the Human Phospho-Kinase Array Kit (R&D Systems) according to the manufacturer's recommendations. Briefly, per condition 1 × 10^7 ^cells were treated for 0, 30, 60, or 240 min. at 37°C as indicated. Lysate (500 μg/condition) was added to the NC-membranes containing spotted Phospho-Kinase (PK) antibodies. Subsequently, secondary Biotin-conjugated anti-Kinase antibodies and Horseradish Peroxidase-conjugated Streptavidin were added to detect the presence of phosphorylated kinases. Between incubation steps, membranes were rigorously washed. Blots were developed by standard chemoluminescence (Roche). Luminescent signals were quantified using the IVIS Spectrum bioluminescent imager (Caliper Biosciences) as photons/sec/sr/cm2. After background luminescence subtraction, the relative kinase activity in treated conditions was calculated by the formula: (PK activity experimental condition/PK activity medium control) × 100%.

### A375M xenograft mouse model

Experiments involving animals were performed in accordance with the experimental protocol approved by the Committee for Research and Animal Ethics of the UMCG. Six week old healthy male athymic mice (n = 8) were purchased from Harlan (Harlan Netherlands B.V., Horst, The Netherlands). Mice were housed in IVC cages and fed ad libitum. Subsequently, mice were subcutaneously inoculated with 2 × 10^6 ^A375M cells suspended in 100 μl matrigel. Tumor growth was monitored daily by electronic caliper measurements. After reaching tumor size of ~ 50 ± 6 mm^3^, mice were randomly assigned into two groups with a sample size of 4. Mice received daily i.v. saline injections or anti-MCSP:TRAIL injections (0.14 mg/kg). After two weeks of treatment, mice were sacrificed by cervical dislocation. Tumor size was calculated by the formula: V = 0,5234 × H × L × W [mm3]. Tumor size was expressed as the percentage of maximum tumor size in Sham-treated mice.

### Liver histopathology

Formalin-fixed liver samples were embedded in paraffin, sectioned into 5-μm thickness and stained with hematoxylin-eosin and microscopically inspected for hepatic tissue damage and inflammation.

### Combination treatment with anti-MCSP:TRAIL and σ-ligands

Cells were plated at 3.0 × 10^4 ^cells/well in a 48-well plate and allowed to adhere overnight. Cells were concurrently treated for 24 h with anti-MCSP:TRAIL with or without rimcazole (15 μM), and (+)pentazocine (200 nM). Synergy was assessed by calculating the cooperativity index (CI) in which the sum of apoptosis induced by single-agent treatment is divided by apoptosis induced by combination treatment. When CI was less than 1, treatment was considered synergistic; when CI equaled 1, treatment was considered additive; when CI was greater than 1, treatment was considered antagonistic.

### Statistical analysis

Data reported are mean values ± standard error of the mean of at least three independent experiments. Statistical analysis was performed by one-way ANOVA followed by Tukey-Kramer post test or by two-sided unpaired Student's t-test. p < 0.05 was defined as a statistically significant difference.

## Competing interests

The authors declare that they have no competing interests.

## Authors' contributions

MB carried out experiments and helped draft the manuscript. AR carried out the *in vivo *experiments and helped draft the manuscript. YW carried out the *in vitro *synergy experiments. MS analyzed and interpreted data. GF contributed essential reagents. RD participated in the coordination of the study. AW participated in the coordination of the study. WH conceived the study and participated in its coordination. EB participated in the design and coordination of the study and drafted the manuscript. All authors read and approved the final manuscript.

## Supplementary Material

Additional file 1 **Figure S1**. **A **A2058 cells were treated with increasing concentrations of anti-MCSP:TRAIL or anti-EpCAM:TRAIL for 16 h and apoptosis was assessed by ∆ψ. **B **A375M cells were treated with 500 ng/mL anti-MCSP:TRAIL for the time-points indicated and caspase-8 activation was assessed. **C **A2058 cells were treated with 500 ng/mL anti-MCSP:TRAIL in the absence or presence of parental MCSP-blocking mAb 9.2.27 or TRAIL-neutralizing mAb 2E5 and caspase-3/-7 activation was assessed. **D **MCSP-restricted binding of anti-MCSP:TRAIL to melanocytes was assessed. Specific binding was demonstrated by pre-incubating melanocytes with mAb 9.2.27 followed by incubation with anti-MCSP:TRAIL. Binding of anti-MCSP:TRAIL was assessed by flow cytometry using a PE-conjugated anti-TRAIL mAb. **E **A2058 and A375M cells were treated with increasing concentrations of anti-MCSP:TRAIL for 16 h and apoptosis was assessed by ∆ψ. Click here for file

Additional file 2 **Table S1**. Table containing the various kinases down regulated by anti-MCSP:TRAIL, their role in oncology and the percentage of inhibition after 1 h treatment with anti-MCSP:TRAIL. Click here for file

Additional file 3 **Figure S2**. Activity of **A **Caspase-8, **B **Caspase-9 was assessed in A375M cells after incubation with anti-MCSP:TRAIL in the presence or absence of rimcazole (15 μM) for 1, 2, 3, 4, 5, 6 or 16 h **C **A375M cells were treated for 16 h with 100 ng/mL of anti-MCSP:TRAIL and/or rimcazole (15 μM) in the presence or absence of zVAD-FMK (20 μM) or zLEHD-FMK and apoptosis was assessed by ∆ψ. Click here for file
