# Supplementary figures and images for: Melanoma-associated Chondroitin Sulfate Proteoglycan (MCSP)-targeted delivery of soluble TRAIL potently inhibits melanoma outgrowth in vitro and in vivo
Source: Mol Cancer. 2010 Nov 23;9:301. doi: 10.1186/1476-4598-9-301 (PMC3000402; doi:10.1186/1476-4598-9-301)

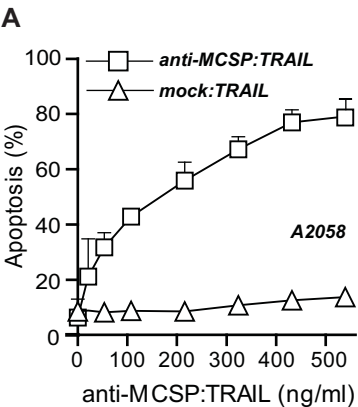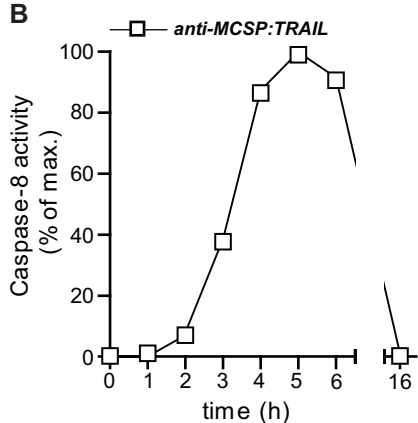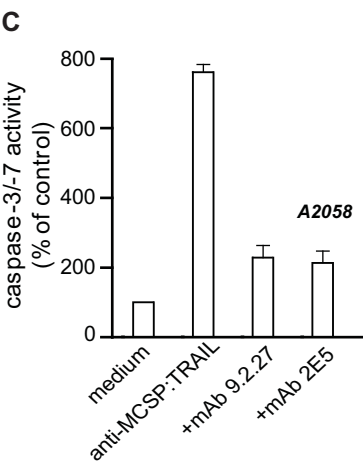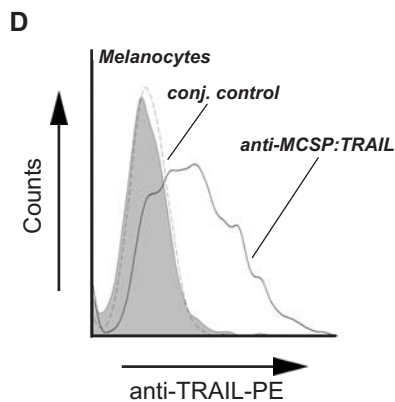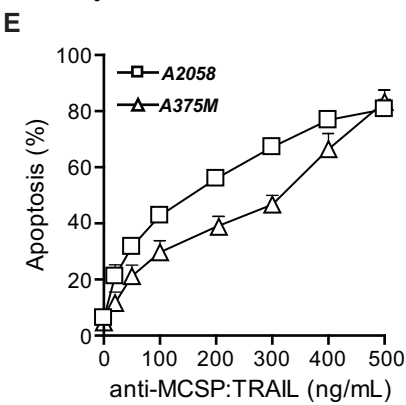

Supplement: Additional file 1 — Figure S1. A A2058 cells were treated with increasing concentrations of anti-MCSP:TRAIL or anti-EpCAM:TRAIL for 16 h and apoptosis was assessed by ∆ψ. B A375M cells were treated with 500 ng/mL anti-MCSP:TRAIL for the time-points indicated and caspase-8 activation was assessed. C A2058 cells were treated with 500 ng/mL anti-MCSP:TRAIL in the absence or presence of parental MCSP-blocking mAb 9.2.27 or TRAIL-neutralizing mAb 2E5 and caspase-3/-7 activation was assessed. D MCSP-restricted binding of anti-MCSP:TRAIL to melanocytes was assessed. Specific binding was demonstrated by pre-incubating melanocytes with mAb 9.2.27 followed by incubation with anti-MCSP:TRAIL. Binding of anti-MCSP:TRAIL was assessed by flow cytometry using a PE-conjugated anti-TRAIL mAb. E A2058 and A375M cells were treated with increasing concentrations of anti-MCSP:TRAIL for 16 h and apoptosis was assessed by ∆ψ. [file 1476-4598-9-301-S1.PDF]

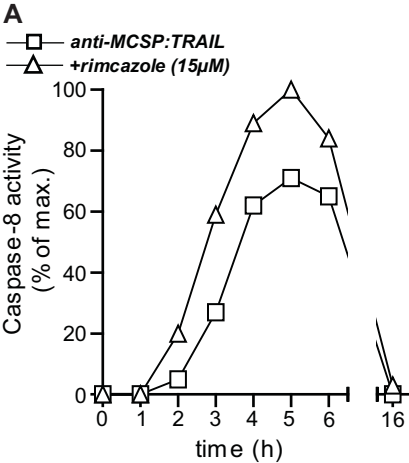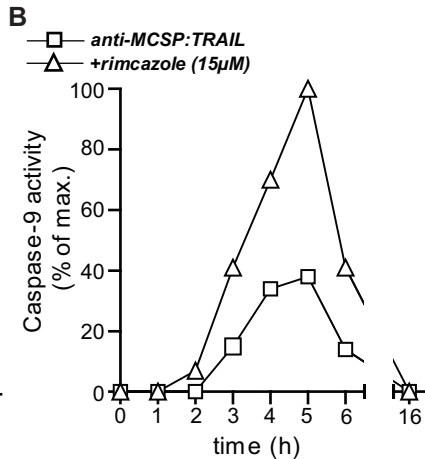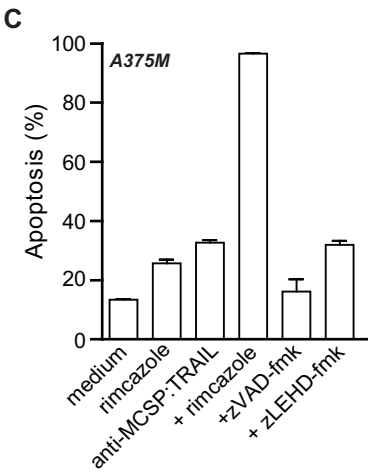

Supplement: Additional file 3 — Figure S2. Activity of A Caspase-8, B Caspase-9 was assessed in A375M cells after incubation with anti-MCSP:TRAIL in the presence or absence of rimcazole (15 μM) for 1, 2, 3, 4, 5, 6 or 16 h C A375M cells were treated for 16 h with 100 ng/mL of anti-MCSP:TRAIL and/or rimcazole (15 μM) in the presence or absence of zVAD-FMK (20 μM) or zLEHD-FMK and apoptosis was assessed by ∆ψ. [file 1476-4598-9-301-S3.PDF]
