# Supplementary material for: Melanoma-associated Chondroitin Sulfate Proteoglycan (MCSP)-targeted delivery of soluble TRAIL potently inhibits melanoma outgrowth in vitro and in vivo
Source: Mol Cancer. 2010 Nov 23;9:301. doi: 10.1186/1476-4598-9-301 (PMC3000402; doi:10.1186/1476-4598-9-301)
Supplement: Additional file 2 — Table S1. Table containing the various kinases down regulated by anti-MCSP:TRAIL, their role in oncology and the percentage of inhibition after 1 h treatment with anti-MCSP:TRAIL. [file 1476-4598-9-301-S2.DOC]

| **Kinase** | **Phosphorylation site** | **scFvMCSP:sTRAIL** | **P-value** | **Function in oncology** |
| --- | --- | --- | --- | --- |
| **Fyn** | Y420 | 69±8 | <0.01 | Src family kinase member involved in integrin-mediated cell adhesion (1;2) |
| **FAK** | Y397 | 75±6 | <0.01 | Focal adhesion kinase involved in cell adhesion and spreading (3-5) |
| **Src** | Y419 | 59±9 | <0.01 | Src family kinase members involved in cell adhesion, invasion, proliferation, survival and angiogenesis during tumor formation (5;6) |
| **Hck** | Y411 | 57±10 | <0.01 |
| **Lyn** | Y397 | 81±12 | <0.05 |
| **Yes** | Y426 | 67±10 | <0.01 |
| **AMPKα1** | T174 | 61±10 | <0.01 | AMP-activated protein kinase involved in energy homeostasis (7;8) |
| **AMPkα2** | T172 | 50±18 | <0.01 | AMP-activated protein kinase involved in energy homeostasis (7;8) |
| **STAT2** | Y689 | 47±17 | <0.01 | Signal transducer and activator of transcription family members involved in cytokine and growth factor signaling (9;10) |
| **STAT3** | Y705 | 43±19 | <0.01 |
| **STAT5a** | Y699 | 55±22 | <0.01 |
| **STAT5b** | Y699 | 55±18 | <0.01 |
| **STAT5a/b** | Y699 | 55±15 | <0.01 |
| **STAT6** | Y641 | 54±19 | <0.01 |
| **β-catenin** | - | 32±8 | <0.01 | Signaling molecule involved in the Wnt pathway (11;12) |
| **p53** | S392 | 56±4 | <0.01 | phosphorylation site involved in nucleolar localization and DNA-binding affinity of p53 (13) |
| **p53** | S46 | 63±9 | <0.01 | phosphorylation site involved in regulation of pro-apoptotic gene transcription (14) |
| **p53** | S15 | 77±7 | <0.01 | phosphorylation site involved in regulation of pro-apoptotic gene transcription (14) |
| **Chk-2** | T68 | 62±3 | <0.01 | Involved in cell cycle control and p53 phosphorylation (15-17) |
| **Jnk** | T183/Y185, T221/Y223 | 59±11 | <0.01 | mitogen-activated protein kinase involved in regulation of apoptosis (18;19) |
| **TOR** | S2448 | 62±21 | <0.05 | Involved in cell cycle control and p53 phosphorylation (14;20) |
| **Erk-1/-2** | T202/Y204, T185/Y187 | 124±48 | n.s. | mitogen-activated protein kinase involved in MCSP-signaling (3;21) |

### Table reference list

(1) Resh M. Fyn, a Src family tyrosine kinase. The International Journal of Biochemistry & Cell Biology 1998 Nov;30(11):1159-62.

(2) Saito YD, Jensen AR, Salgia R, Posadas EM. Fyn: a novel molecular target in cancer. Cancer 2010 Feb 11.

(3) Yang J, Price MA, Neudauer CL, Wilson C, Ferrone S, Xia H, et al. Melanoma chondroitin sulfate proteoglycan enhances FAK and ERK activation by distinct mechanisms. J Cell Biol 2004 Jun 21;165(6):881-91.

(4) Schwock J, Dhani N, Hedley DW. Targeting focal adhesion kinase signaling in tumor growth and metastasis. Expert Opin Ther Targets 2010 Jan;14(1):77-94.

(5) Zhao J, Guan JL. Signal transduction by focal adhesion kinase in cancer. Cancer Metastasis Rev 2009 Jun;28(1-2):35-49.

(6) Kim L, Song L, Haura E. Src kinases as therapeutic targets for cancer. Nat Rev Clin Oncol 2009 Oct;6(10):587-95.

(7) Koh H, Chung J. AMPK links energy status to cell structure and mitosis. Biochemical and Biophysical Research Communications 2007 Nov 3;362(4):789-92.

(8) Shaw RJ. Glucose metabolism and cancer. Curr Opin Cell Biol 2006 Dec;18(6):598-608.

(9) Yu H, Pardoll D, Jove R. STATs in cancer inflammation and immunity: a leading role for STAT3. Nat Rev Cancer 2009 Nov;9(11):798-809.

(10) Lopez-Bergami P, Fitchman B, Ronai Z. Understanding signaling cascades in melanoma. Photochem Photobiol 2008 Mar;84(2):289-306.

(11) O'Connell MP, Weeraratna AT. Hear the Wnt Ror: how melanoma cells adjust to changes in Wnt. Pigment Cell Melanoma Res 2009 Dec;22(6):724-39.

(12) Moon R, Kohn A, Ferrari G, Kaykas A. WNT and [beta]-catenin signalling: diseases and therapies. Nat Rev Genet 2004 Sep;5(9):691-701.

(13) Karni-Schmidt O, Friedler A, Zupnick A, McKinney K, Mattia M, Beckerman R, et al. Energy-dependent nucleolar localization of p53 in vitro requires two discrete regions within the p53 carboxyl terminus. Oncogene 2007 Jan 22;26(26):3878-91.

(14) Castedo M, Ferri KF, Blanco J, Roumier T, Larochette N, Barretina J, et al. Human immunodeficiency virus 1 envelope glycoprotein complex-induced apoptosis involves mammalian target of rapamycin/FKBP12-rapamycin-associated protein-mediated p53 phosphorylation. J Exp Med 2001 Oct 15;194(8):1097-110.

(15) Shieh S, Ahn J, Tamai K, Taya Y, Prives C. The human homologs of checkpoint kinases Chk1 and Cds1 (Chk2) phosphorylate p53 at multiple DNA damage-inducible sites. Genes & Development 2000 Feb 1;14(3):289-300.

(16) Stracker TH, Usui T, Petrini JH. Taking the time to make important decisions: the checkpoint effector kinases Chk1 and Chk2 and the DNA damage response. DNA Repair (Amst) 2009 Sep 2;8(9):1047-54.

(17) Stracker TH, Couto SS, Cordon-Cardo C, Matos T, Petrini JH. Chk2 suppresses the oncogenic potential of DNA replication-associated DNA damage. Mol Cell 2008 Jul 11;31(1):21-32.

(18) Lin A, Dibling B. The true face of JNK activation in apoptosis. Aging Cell 2002 Dec;1(2):112-6.

(19) Dhanasekaran DN, Reddy EP. JNK signaling in apoptosis. Oncogene 0 AD;27(48):6245-51.

(20) Hay N, Sonenberg N. Upstream and downstream of mTOR. Genes & Development 2004 Aug 15;18(16):1926-45.

(21) Russo AE, Torrisi E, Bevelacqua Y, Perrotta R, Libra M, McCubrey JA, et al. Melanoma: molecular pathogenesis and emerging target therapies (Review). Int J Oncol 2009 Jun;34(6):1481-9.
